# Growth, Nutrient Deposition, Plasma Metabolites, and Innate Immunity Are Associated with Feeding Rate in Juvenile Starry Flounder (*Platichthys stellatus*)

**DOI:** 10.3390/ani14213127

**Published:** 2024-10-30

**Authors:** Jeong-Hyeon Cho, Ali Hamidoghli, Sang-Woo Hur, Bong-Joo Lee, Seunghan Lee, Kang-Woong Kim, Seunghyung Lee

**Affiliations:** 1 Subtropical Fisheries Research Institute, National Institute of Fisheries Science, Jeju 61610, Republic of Korea; cjh.jan23@gmail.com; 2 Aquaculture Research Institute, University of Idaho, Hagerman, ID 83332, USA; 3 Aquafeed Research Center, National Institute of Fisheries Science, Pohang 37517, Republic of Korea; 4 Department of Aqualife Medicine, Kongju National University, Yesan 32439, Republic of Korea; 5 Department of Aquaculture and Aquatic Science, Kunsan National University, Gunsan 54150, Republic of Korea; 6 Major of Aquaculture and Applied Life Sciences, Division of Fisheries Life Sciences, Pukyong National University, Busan 48513, Republic of Korea

**Keywords:** starry flounder, feeding rate, nutritional status, innate immunity, broken line

## Abstract

A 10-week feeding trial was conducted to evaluate the effects of different feeding rates on growth performance, nutrient deposition, plasma metabolite, and immunity of juvenile starry flounder. Fish (initial mean body weight, 183.6 ± 2.3 g) were subjected to eight feeding rates (0.4, 0.8, 1.2, 1.6, 2.0, 2.4, 2.8, and 3.2% body weight/day [BW/d]) with a commercial diet containing 53.5% crude protein and 10.2% crude lipid. After the feeding trial, fish growth increased significantly (*p* < 0.05) from 0.4% to 2.0% BW/d, with no significant differences being observed beyond 2.0% BW/d. Protein and lipid gains in the whole body and liver of the fish fed 2.0–3.2% BW/d were significantly (*p* < 0.05) higher than those of the fish fed 0.4% and 0.8% BW/d. Conversely, protein retention in the whole body and the liver decreased with an increased feeding rate. Lysozyme activity was significantly (*p* < 0.05) higher in the fish fed 1.6–2.8% BW/d than in those fed 0.4–1.2% BW/d. The best-fit model analyses for optimum feeding rate (OFR) revealed that the estimate for each parameter varied between 0.7% (feed conversion ratio) and 3.1% (lipid gain in carcass) BW/d. The OFR for productivity (weight gain) and enhanced innate immunity (lysozyme) were estimated at 2.4% and 1.7% BW/d, respectively.

## Simple Summary

Starry flounder (*Platichthys stellatus*), a marine flatfish, is a key species in marine aquaculture due to its high market price and tolerance to a wide range of salinities; however, limited information on optimal feeding in this species has hindered efforts to improve its aquaculture productivity. This study used eight feeding rates, ranging from 0.4% to 3.2% body weight per day, over a 10-week period, to determine optimal feeding rate (OFR) in starry flounder. The results of the regression analysis showed that feeding rates between 1.6% and 2.4% body weight per day optimized growth, nutrient deposition, and immune function. The OFR for growth was determined to be 2.4%, while the OFR for enhancing innate immunity was estimated at 1.7%. These findings suggest that adjusting feeding rates is essential for improving productivity and immune response in starry flounder aquaculture.

## 1. Introduction

The extensive growth in global aquaculture production has highlighted its potential as a source of healthy protein for the growing human population in the future [1]. However, sustainable development of aquaculture faces environmental, economic, and social challenges [2]. One such challenge is supplying adequate amounts of nutrient-balanced diets that can support the growth and immune system of fish while minimizing the exploitation of natural resources [3]. Feed is an important aspect of aquaculture development, as it accounts for a large portion of the production costs [4]. Therefore, feeding management has a significant impact on the economic feasibility of a production system and, to a large extent, on the sustainability of aquaculture. Feeding rate and frequency strongly influence not only growth and immunity but also various other parameters of fish, including nutritional quality, digestive enzyme activity, and plasma metabolites [5,6,7].

Among these factors, the feeding rate directly affects fish growth, feed utilization efficiency, and survival of cultured organisms. Inadequate supply of feed can cause growth retardation and high mortality in fish. In particular, overfeeding leads to increased feed costs and general deterioration of water quality, which can consequently reduce the profitability of aquaculture [8]. Determining an adequate feeding rate for fish is of fundamental importance for commercial aquaculture production of fish, and results obtained in such studies directly bring about significant improvements in the profitability of aquaculture [8,9]. The optimum feeding rate (OFR) is defined as the lowest amount of feed, usually below the satiety level, that results in the highest growth yield [10]. Feeding rates below the optimum level can result in poor growth and weakened immune responses [11,12]. This can negatively affect production operations and result in financial problems [13]. Conversely, a higher feeding rate results in waste of feed, deterioration of water quality, spread of pathogenic diseases, and reduced immunity [6,14,15], which puts a financial burden on the producers [16]. Therefore, maintaining an optimal feeding rate is critical to the success of aquaculture production systems.

Several studies have focused on the effects of different feeding rates on fish growth performance. Studies on rockfish *Sebastes schlegeli* [17], green sturgeon *Acipenser medirostris* [18], Atlantic salmon *Salmo salar* [15], white sturgeon *Acipenser transmontanus* [19], Brazilian sardine *Sardinella brasiliensis* [5], olive flounder *Paralichthys olivaceus* [20], and Nile tilapia *Oreochromis niloticus* [21] have clearly demonstrated the effects of suboptimal feeding rates on fish growth and body composition. However, understanding of the immune responses to varying feeding rates is limited. Optimum immune function in fish is maintained by the consumption of nutrients and energy in the diet. If adequate nutrition is not received, the immune system cannot defend the host against pathogens because it is deprived of the required resources [22]. Nutrient deficiency impairs disease resistance and alters immunocompetence [23]. In general, lysozyme, heat shock protein 70 (HSP-70), and glutathione peroxidase (GSH-PX) activities have been utilized as important parameters of non-specific immunity in fish [23,24,25]. It was reported that stress factors such as stocking density, water pollution, and nutrition affect immunity and antioxidant activity [25,26,27], but there are few reports on the changes in immunity that are brought about due to feeding rate. Additionally, immunoglobulin M (IgM) is an important immunoglobulin found in teleosts, which are more dependent on IgM for the functioning of their immune systems than other marine species [28]. Superoxide dismutase (SOD) is an antioxidant enzyme that plays an important role in the immune system and removes superoxide anions from tissues [29]. As one of the strategies to increase aquaculture productivity, the health status of fish according to feeding rate should also consider changes in the immunity of fish due to insufficiency and overfeeding of feed. Lee et al. [6] reported that the feeding rate had a significant influence on the nutritional status of olive flounder, whereas underfeeding reduced the expression of immune-related genes. Antimicrobial polypeptides are significantly decreased at suboptimal feeding rates in hybrid striped bass, resulting in increased disease susceptibility [11]. These aforementioned studies have investigated the OFR for different fish species at specific life stages and revealed that OFR is highly dependent on species type, life stage, and culture conditions.

Starry flounder *Platichthys stellatus* is a marine flatfish distributed from South Korea and southern Japan to the northwestern territories of Canada and the USA. Its aquaculture production in South Korea is approximately 4353 tons, with an approximate value of USD 50,000 [30]. Starry flounder is a valuable aquaculture species owing to its high market value and tolerance to a wide range of salinities. However, the nutritional management of starry flounder has scarcely been studied [31,32]. Therefore, the aim of this study was to investigate the effects of graded feeding rates on growth and biological indices, body composition, plasma metabolites, lysozyme, interleukin 1β (IL-1β), IgM, HSP-70, GSH-PX, and SOD in starry flounder, and to determine the OFR based on one-slope straight broken-line, two-slope straight broken-line, quadratic broken-line, and quadratic models.

## 2. Materials and Methods

All animal care and standard operating procedures involving animal ethical considerations, including anesthesia, dissection, and euthanasia, were approved by the Institutional Animal Care and Use Committee of the National Institute of Fisheries Science (NIFS), Republic of Korea, and conducted in accordance with the Guidelines for Experimental Animals (2019-NIFS-IACUC-13).

### 2.1. Fish Maintenance and Feeding Trial

Starry flounder reared in the Fisheries Resources Institute, Gyeongsangbuk-do, located near our research facility (approximately 45 km away), were stocked in 8000-L polyethylene circular tanks (diameter, 3.5 m; height, 0.8 m) during the accumulation period. The fish were hand-fed a commercial feed (Suhyup Feed Co., Gyeongsangnam-do, Republic of Korea; particle size: 5.0–5.3 mm) produced for starry flounder, twice a day. The feed comprised sinking pellets that contain animal protein sources including fish meal, >69%; plant protein sources including soybean meal, <11%; wheat flour, <17%; and fish oil, >2%. The nutrient composition of the feed analyzed using the AOAC method [33] was 5.8% moisture, 53.5% crude protein, 10.2% crude lipid, and 13.8% crude ash. Starry flounder (*n* = 720) were randomly distributed into 24 circular polyethylene tanks (diameter, 1.3 m; height, 0.8 m; volume, 1000 L), at a stocking density of 30 fish per tank (body weight: 183.6 ± 2.3 g, mean ± standard deviation). The tanks were operated in a flow-through configuration with seawater at a rate of 10 L/min and equipped with an aeration apparatus. Feed availability was manipulated by allocating eight feeding rates (0.4, 0.8, 1.2, 1.6, 2.0, 2.4, 2.8, and 3.2% body weight per day; BW/d) to three tanks. The feeding trial lasted ten weeks. The average body weight of each treatment group was measured every two weeks during the feeding trial and feed supply amount was changed every two weeks based on fish growth. The fish were hand-fed twice daily (at 09:00 and 17:00) according to the specified feeding rate. The feeding trial was conducted indoors, and the photoperiod was set to 12 h of light and 12 h of dark. Seawater temperature was monitored daily using a temperature data logger (HOBO^®^ Water Temp Pro v2 (U22-001); ONSET, Bourne, MA, USA) and ranged from 12.7 °C to 20.1 °C (September to November). Dissolved oxygen level and pH were measured daily using a YSI PRO 1020 multi-parameter meter (YSI Inc., Yellow Springs, OH, USA) and maintained at 8.2 ± 0.6 mg/L and 8.1 ± 0.2, respectively, throughout the trial.

### 2.2. Measurements

#### 2.2.1. Growth Performance and Chemical Analysis

After the 10-week growth trial, all fish in each tank were weighed to calculate weight gain (WG, %), specific growth rate (SGR, %/day), thermal growth coefficient (TGC), feed intake (FI, g/fish), and feed conversion ratio (FCR). Five fish from each tank were randomly selected and euthanized with an overdose of 2-phenoxyethanol (200 parts per million; Sigma-Aldrich, St. Louis, MO, USA), measured for individual total length (cm) and body weight (g), and used to calculate the condition factor (CF, g/cm^3^). The fish were then dissected, and the weight of the liver, viscera (without spleen and gallbladder), and dissected fish were measured to determine the hepatosomatic index (HSI), viscerosomatic index (VSI), and protein and lipid gains. These indices were used to measure biological indices. The collected liver, viscera, and the dissected fish were stored at –20 °C for proximate analysis. The calculation for each measurement is as follows [34]:

WG (%) = [final wet weight (g/fish) − initial wet weight (g/fish)]/initial wet weight (g/fish) × 100

SGR (%/day) = [Ln final wet weight (g/fish) − Ln initial wet weight (g/fish)]/number of days × 100

TGC = [final wet weight (g/fish)^1∕3^ − initial wet weight (g/fish)^1∕3^] × (sum day degrees Celsius)^−1^ × 1000

FCR = dry feed intake (g/fish)/wet weight gain (g/fish)

CF = [wet weight (g)/total length (cm)^3^] × 100

HSI (%) = [wet weight of liver (g)/wet weight (g)] × 100

VSI (%) = [wet weight of viscera (g)/wet weight (g)] × 100

After final weighing and prior to final sampling, feed was withheld from the fish for 24 h. Five additional fish were randomly captured and euthanized with an overdose of 2-phenoxyethanol (200 ppm) to collect blood using a needle, heparinized disposable syringe (3 mL/cc), and vacutainer (BD Vacutainer^®^ Ref #36664; Franklin Lakes, NJ, USA) through caudal vein puncture. The collected blood was centrifuged at 7168× *g* (VS-24SMTi; VISION Scientific, Co., Ltd., Daejeon, Republic of Korea) for 20 min at 4 °C to separate the plasma. The plasma samples were stored at −80 °C for subsequent analyses of stress and immune response parameters, such as aspartate aminotransferase (AST), alanine aminotransferase (ALT), triglycerides, cholesterol, glucose, total protein, lysozyme, IL-1β, IgM, HSP-70, GSH-PX, and SOD. Plasma metabolites were determined with commercially available kits (ALT, Product Code: 981769; AST, Product Code: 981771; triglycerides, Product Code: 981786; cholesterol, Product Code: 981813; glucose, Product Code: 981780; total proteins, Product Code: 981827; Thermo Fisher Scientific Korea, Ltd., Seoul, Republic of Korea) using blood analyzer (Indiko^TM^; Thermo Fisher Scientific Korea, Ltd., Seoul, Republic of Korea). Plasma lysozyme, IL-1β, IgM, HSP-70, GSH-PX, and SOD levels were measured using an enzyme-linked immunosorbent assay (ELISA) microplate reader (SYNERGY H1; BioTek Instruments, Inc., Winooski, VT, USA) and commercial kits (Cat. #E17296Fh for lysozyme, E13259Fh for IL-1β, E12045Fh for IgM, E16327Fh for HSP-70, E15930Fh for GSH-PX, and E15929Fh for SOD; CUSABIO, Wuhan, Chian). The assays were performed according to the manufacturer’s protocol.

For calculating gain and nutrient retention, 30 fish at the beginning and 5 fish from each tank at the end of the feeding trial were subjected to sample collection for analysis of nutrient content. Gain and nutrient retention in the whole body, carcass, liver, and viscera of fish were calculated using the following equations:

[(FBW or FTW × FNC) − (IBW or ITW × INC)]/100

[(FBW or FTW × FNC) − (IBW or ITW × INC)]/nutrient feed (g)

where FBW, FTW, FNC, IBW, ITW, and INC are final body weight (g), final tissue weight (g), final nutrient composition (%), initial body weight (g), initial tissue weight (g), and initial nutrient composition (%), respectively.

Nutrient content, including moisture, crude protein, crude lipid, and crude ash, was analyzed using standard methods [33]. The moisture content was determined by drying the sample in an oven (OF-W155; Daihan Scientific Co., Ltd., Daegu, Republic of Korea) at 135 °C for 2 h. Crude protein was measured using the Kjeldahl method (Gerhardt VAP 50 OT/TT125; Königswinter, Germany). A nitrogen conversion factor of 6.25 was applied to convert the amount of nitrogen detected into the protein content. Crude lipids were determined using lipids extracted with ethyl ether (Soxtec 2043; Foss, Hillerød, Denmark). The crude ash content was determined using a muffle furnace (FHPX-14; Daihan Scientific Co., Ltd., Daegu, Republic of Korea) at 600 °C for 6 h. All biochemical analyses were performed in triplicate.

#### 2.2.2. Determination of Optimum Feeding Rate

Broken line or quadratic regression model analyses that model the dose–response relationship between dependent and independent variables are commonly used to determine an estimate predicted to give the best response [19,35,36,37,38,39]. The broken-line model, encompassing the one-slope straight broken-line model (one-slope BL model), two-slope straight broken-line model (two-slope BL model), and quadratic broken-line model (quadratic BL model), identifies a single breakpoint. This breakpoint represents the intersection of linear and plateau lines (one-slope BL model), linear and positively or negatively sloped lines (two-slope BL model), and quadratic and plateau lines (quadratic BL model) (see [36] for details). The breakpoint indicates the optimum feed rate (OFR) at which measurements such as growth and feed efficiency are predicted to reach their maxima. Additionally, the quadratic model, a second-order polynomial, predicts OFR as the vertex of the polynomial curve. We evaluated the performance of these models by selecting the best-fit model for the dataset obtained in this study. Model performance was assessed using the adjusted coefficient of determination (R^2^_adj_) and corrected Akaike information criterion (AICc), where larger R^2^_adj_ and smaller AICc values indicate better model performance. The statistical software R 3.0.1 [40] was used for the analysis.

### 2.3. Statistical Analysis

The results were analyzed using IBM SPSS 19 software package for Windows (SPSS Inc., Chicago, IL, USA). Data were evaluated for assumptions, including normality and homogeneity of variance, using the Shapiro–Wilk and Levene’s tests, respectively, and no violations were detected (*p* > 0.05). Statistical analyses were conducted using one-way analysis of variance (ANOVA) with a 95% significance level (*p* < 0.05). When a significant treatment effect was detected, Tukey’s honest significant difference (HSD) test was used to assess significant differences among means.

## 3. Results

### 3.1. Growth and Biological Indices

The growth and biological indices of starry flounder fed at various feeding rates for 10 weeks are shown in Table 1. The FBW, WG, SGR, and TGC of the starry flounder significantly (*p* < 0.05) increased steadily from 0.4% to 2.0% BW/d. No significant differences were observed in the FBW, WG, SGR, and TGC of the starry flounder from 2.0% to 3.2% BW/d (*p* > 0.05). In contrast, the FCR of fish fed 0.8 and 1.2% BW/d was significantly lower than that of the other groups. Fish fed at higher feeding rates (2.4–3.2% BW/d) had FCR values greater than 1.5 BW/d, which was significantly higher than that of fish fed at lower feeding rates (0.4–2.0% BW/d). Biological indices showed a slightly increasing trend for the CF of starry flounder fed at different feeding rates, although there were no significant differences across feeding rates of 0.8–3.2% BW/d (*p* > 0.05). The HSI of fish fed 2.0 and 2.8% BW/d was significantly higher than that of all other groups (*p* < 0.05). There were no significant differences in the VSI and survival among the treatment groups.

### 3.2. Proximate Compositions

Proximate composition analyses of the whole body, carcass, liver, and viscera of starry flounder fed at various feeding rates for 10 weeks are shown in Table 2. The whole-body crude lipid content of starry flounder showed an increasing trend, corresponding to a gradual increase in feeding rates up to 2.0% (*p* < 0.05). At 2.0–3.2% BW/d, the whole-body crude lipid content remained consistent (*p* > 0.05). The whole-body crude ash contents of fish fed 0.4 and 0.8% BW/d were significantly higher than those of fish fed 1.2–3.2% BW/d. The carcass moisture content of the starry flounder also showed a decreasing trend with increasing feeding rates, with significant differences between the 0.4–1.2 and the 2.4–3.2% BW/d (*p* < 0.05). Carcass protein content was not greatly influenced by the various feeding rates, and significant differences were only observed between the lowest (0.4 and 0.8% BW/d) and the highest (3.2% BW/d) feeding rates. The crude lipid content of carcasses was significantly higher at higher feeding rates (1.6–3.2% BW/d) than at the lowest feeding rate (0.4% BW/d). Liver moisture and crude protein showed similar trends: both increased up to 1.6% BW/d and then remained unchanged. In contrast, the liver crude lipid levels suddenly increased at 2.0% BW/d and remained unchanged thereafter. In addition, visceral lipid content was higher at 1.6–2.4% BW/d than at 0.4–1.2% BW/d (*p* < 0.05).

### 3.3. Plasma Metabolites

Plasma metabolites (AST, ALT, triglycerides, cholesterol, glucose, and total protein) of starry flounder fed at various feeding rates for 10 weeks are listed in Table 3. Plasma triglyceride levels were significantly higher in fish fed the 1.6–3.2% BW/d than in those fed 0.4 and 0.8% BW/d (*p* < 0.05). In the case of cholesterol, there was no substantial difference among the dietary treatments, and only the 0.4% BW/d group had significantly lower cholesterol levels than the 0.8, 1.6, 2.0, 2.4, 2.8, and 3.2% BW/d groups. The total protein showed the same trend, with the lowest values in the lowest feeding rate (0.4% BW/d) group. There were no significant differences in plasma AST, ALT, or glucose levels among dietary treatments (*p* > 0.05).

### 3.4. Protein and Lipid Gains and Retentions

Protein and lipid gains and retentions in the whole body, carcass, liver, and viscera of starry flounder fed at various feeding rates for 10 weeks are presented in Table 4. The whole-body protein and lipid gains of fish fed 2.0–3.2% BW/d were significantly higher than those of fish fed 0.4–1.2% BW/d (*p* < 0.05). A steady upward trend was observed for carcass protein and lipid gains, which were significantly higher in fish fed 2.0–3.2% BW/d than in fish fed 0.4–1.2% BW/d (*p* < 0.05). Liver protein and lipid gains were also significantly higher in fish fed 2.0–3.2% BW/d than in fish fed 0.4 and 0.8% BW/d. The same trend was observed for visceral protein and lipid gain.

In terms of nutrient retention, whole body, carcass, liver, and visceral protein levels decreased with increasing feeding rate. Whole-body lipid retention was significantly higher in fish fed 0.8–1.6% BW/d than in fish fed 0.4, 2.4, 2.8, and 3.2% BW/d (*p* < 0.05). Carcass lipid retention followed the same trend, with 0.8 and 1.2% BW/d having significantly higher lipid retention than 0.4 and 3.2% BW/d. Although this trend was not observed for liver lipid retention, fish fed 0.4% BW/d showed the lowest values, and no significant differences were noted among the other groups. Visceral lipid retention was significantly higher in fish fed 0.8–2.0% BW/d than in those fed 0.4%, 2.8%, and 3.2% BW/d (*p* < 0.05).

### 3.5. Optimum Feeding Rate

**Figure 1:**
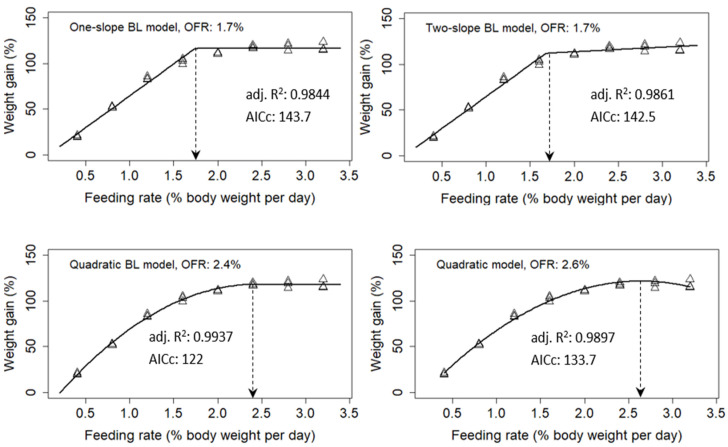
Line/curve fits to the observations obtained from starry flounder (initial body weight: 183.6 ± 2.3 g) fed at various feeding rates (% body weight/day). The fitting was performed using the one-slope straight broken-line (One-slope BL model; (**top left**)), two-slope straight broken-line (Two-slope BL model; (**top right**)), quadratic broken-line (Quadratic BL model; (**bottom left**)), and quadratic (Quadratic model; (**bottom right**)) models. The triangle symbol indicates the growth response, weight gain (%), responding to the respective feeding rate. The pointing arrow indicates the optimum feeding rate (OFR) estimated by each model. The model selection criteria, including R^2^adj (adjusted coefficient of correlation) and AICc (corrected Akaike information criterion), were calculated for the selection of best-fit model among the tested models. Larger R^2^adj and smaller AICc values indicate the better performance of a model.

The OFR of starry flounder determined based on various measurements are listed in Table 5. Among the various feeding rates, the OFR was estimated based on one-, two-, and quadratic broken-line and quadratic models. Among all the analyzed models, the best-fit model appeared when R^2^adj was the highest and AICc was the lowest. Regarding WG, the quadratic broken-line model had the highest R^2^adj and lowest AICc and seemed to be the best-fit model. Therefore, according to the quadratic broken-line model, the OFR of starry flounder was 2.4% BW/d (Figure 1). The OFR for whole-body protein gain was 1.5% BW/d based on the two-slope broken-line model, but the OFR for whole-body lipid gain was 2.3% BW/d based on the quadratic broken-line model. The OFR for higher lysozyme activity was 1.7% BW/d, based on the two-slope broken-line model (Table 5, Supplementary Materials).

### 3.6. Plasma Immune-Related Parameters

**Figure 2:**
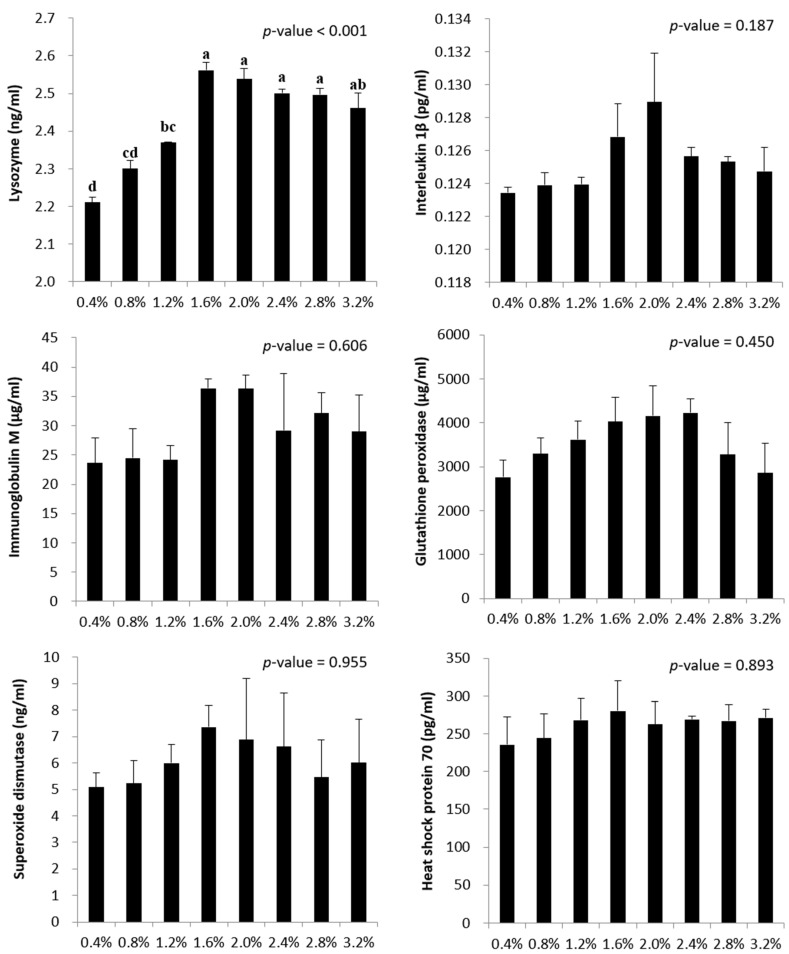
Levels of plasma lysozyme, IL-1β, IgM, GSH-PX, SOD, and HSP-70 in starry flounder fed at various feeding rates (% body weight/day) for 10 weeks. Statistical differences in the concentration (mean ± standard errors) within the interaction effect (*N* = 3 tanks; 5 fish/tank) in response to the different feeding rates were analyzed using one-way analysis of variance (ANOVA). When significance (*p* < 0.05) was detected, multiple range test using Tukey’s HSD test was performed. (etters indicating a significant difference.).

Plasma lysozyme, IL-1β, IgM, HSP-70, GSH-PX, and SOD levels in starry flounder fed at various feeding rates for 10 weeks are shown in Figure 2. Lysozyme activity was significantly higher in fish fed 1.6–2.8% BW/d than in fish fed 0.4–1.2% BW/d (*p* < 0.05). However, there were no significant differences in lysozyme activity of fish fed at feeding rates of 1.6–3.2% (*p* > 0.05). No significant differences were observed in plasma IL-1β, IgM, HSP-70, GSH-PX, and SOD levels in starry flounder fed at different feeding rates (*p* > 0.05).

## 4. Discussion

A strong relationship exists between feeding rate and growth performance in fish [6,8,19,20,41]. In the present study, growth performance parameters such as FBW, WG, and SGR clearly increased at a feeding rate of 2.0% and then remained unchanged. Lee et al. [6] reported that the FBW and WG of juvenile olive flounder increased with increasing feeding rates up to a certain point (10% feeding rate), and then plateaued. A similar trend was observed for the juvenile hybrid sturgeon *Acipenser schrenckii* × *Acipenser baerii* with enhanced FBW when the feeding rate increased from 2% to 4% and then did not change from 4% to 5% feeding rates [9]. This shows that underfeeding results in growth depression, and that overfeeding does not necessarily improve the growth performance of fish. Underfeeding causes high competition, resulting in uneven feed consumption, higher energy expenditure, and subsequent growth depression [15,42]. In contrast, overfeeding might maximize growth but will lead to water quality deterioration and increased production costs [43,44]. Therefore, it is important to determine the OFR of the target species.

In the present study, four statistical models were compared to determine the OFR for starry flounder based on weight gain. According to the model selection criteria (R^2^adj and AICc), the quadratic broken-line model showed an optimal feeding rate of 2.4%. The estimation of optimum levels in nutritional studies has been primarily based on regression models, compared to the simple ANOVA multiple range tests, owing to their suitability for dose–response studies [19,37,39]. However, the application of a single model with the best-fit over multiple models can be advantageous in obtaining an accurate estimation. This is because of the unique specifications of each experimental design, which require the selection of the best-fit model, among others [39]. Studies on olive flounder [6,20], white sturgeon [19], and lake sturgeon [45] have shown that the best-fit model for determining the OFR is a quadratic broken line. Previous studies have used the two-slope broken-line model to determine OFR in other species [12,46]. To the best of our knowledge, this is the first study to evaluate the OFR of starry flounder. Generally, OFRs are highly dependent on species type, size, and rearing environment [8,47]; therefore, comparisons with previous studies might not draw a solid conclusion. Further studies on the OFR of starry flounders of different sizes are required.

Fish body composition is an indicator of fillet quality from a human consumption perspective and is influenced by feeding rate [15]. In the present study, the crude lipid content of the whole body, carcass, liver, and viscera of starry flounder increased with increasing feeding rates and remained unchanged or even decreased at higher feeding rates in the case of the liver and viscera. This correlated with the lipid gain and retention results presented in Table 4. Similar results were observed for white sturgeons [12,19], Brazilian sardines [5], olive flounder [20], and Atlantic salmon [15]. Generally, a higher feed consumption means a higher energy intake that can be converted into fat in the fish body [48]. However, the rate of nutrient accumulation decreased with increasing feeding rate as the fish approached satiation [19]. Previous studies have shown insignificant changes in fish body composition when optimum or higher OFRs are provided [49]. This explains the fluctuations in lipid content observed in the present study. Our results also showed a typical correlation between moisture and lipid content and carcass composition, which was reported in several previous studies [12,18,19,49,50]. This inverse relationship between lipids and moisture could be related to the tendency of animal cells to maintain their size by replacing lost organic matter with water [51]. Our observations did not reveal any visible changes in the crude protein content of the whole body, carcass, or viscera. However, in the liver, lower feeding rates resulted in a higher crude protein content. The liver quickly responds to feed withdrawal in animals, as it decreases in size (proven by our HSI results) with fish weight loss and increases in protein degradation [52]. Protein degradation releases amino acids that compensate for the energy loss that occurs during low feeding rates and/or starvation. Animals, including fish, are designed to meet the energy requirements of their body reserves when feed is scarce [53]. This may explain the increased protein and/or amino acid contents in the liver at low feeding rates. The same trend was observed in Table 4, which shows liver protein retention. Protein and lipid gains for the whole body, carcass, liver, and viscera showed an ascending trend based on the increased feeding rate, which correlates with our results for growth performance. Similar results were reported in previous studies [19,54], showing that the weight increase in starry flounder, in response to the feeding rate, is associated with both proteins and lipids.

Among the plasma metabolites investigated in this study, only triglycerides, cholesterol, and total protein showed increasing trends with increasing feeding rates. ALT, AST, and glucose levels were not significantly affected by the feeding rate. Similar results were observed in common carp *Cyprinus carpio* [55], Brazilian sardine [5], and white sturgeon [19]. Plasma metabolites are accurate representatives of the nutritional status of fish but are also affected by factors such as stress, hormones, and metabolism [51,56]. Triglycerides are the main form of lipid storage and energy in animals [57], and cholesterol is a type of lipid and an important metabolic precursor for several compounds, such as hormones and lipoproteins [58]. Feed deprivation results in reduced energy and lipid storage, with most of the energy invested in maintaining metabolic homeostasis [59]. This could explain our observation of reduced triglyceride and cholesterol levels at the lowest feeding rates. Moreover, as previously mentioned, reduced feed intake results in protein catabolism for energy purposes, which can cause a reduction in total serum protein [51]. Shimeno et al. [55] concluded that at low feeding rates, the regulation of nutrient metabolism is more towards halting lipogenesis and glycolysis and maintaining glycogenesis and protein degradation for energy and blood glucose supplementation. This is consistent with our observation that serum glucose levels were not significantly affected even at the lowest feeding rate.

Most studies evaluating OFR in fish have focused on growth performance [9,12,21,36], and few studies have evaluated the effects of feeding rate on fish immune responses. Understanding the changes in non-specific immune responses (e.g., lysozyme, IgM, and SOD) of fish according to the feeding rate is crucial for sustainable aquaculture production, as it can help prevent fish diseases and increase survival rates. Lysozyme, an enzyme capable of lysing pathogenic bacterial cells, serves as a major indicator of non-specific immune responses [24]. SOD, an antioxidant enzyme, plays an important role in the immune system by removing superoxide anions from tissues [29]. IgM is an important immunoglobulin found in teleost fish and relies more on IgM than other marine species [28]. The results of the present study showed significantly higher serum lysozyme activity in fish provided with diets at feeding rates of 1.6–2.8%. This corresponds to the results obtained for the growth performance and OFR. In contrast, changes in serum IgM and SOD levels were not significant. Consistent with our results, Guo et al. [60] observed increased lysozyme activity in Dolly Varden trout *Salvelinus malma* fed at the optimum feeding frequency. Li et al. [61] also reported higher lysozyme activity when blunt snout bream *Megalobrama amblycephala* was fed at a higher rate. Nutrition is one of the main factors influencing lysozyme activity in fish, as nutrients provide building blocks for innate cellular and humoral immunity [24]. Apart from protein and energy, malnutrition of individual micronutrients such as vitamins (e.g., A, D, and E) and minerals (e.g., zinc, iron, and selenium) can negatively influence fish immune function [62].

IL-1β is known to play a role in tumorigenesis owing to its immunomodulatory properties, regulation of the intestinal microbiota, and influence on differentiation and apoptosis [63]. GSH-PX has a wide range of antioxidant abilities in the body [64], while HSP-70 protects cells from oxidative stress [65]. In the present study, there was no significant difference in IL-1β, GSH-PX, and HSP-70 levels according to feeding rate. Since the survival rate of all experimental groups was 100% during the feeding period, it can be concluded that the feeding rate does not affect the antioxidant, immunity, or stress responses. However, few studies have confirmed the effect of the feeding rate on fish immunity. In a study by Lee et al. [6], downregulation of immune-related genes, such as IL-8 and IgM, was clearly observed in olive flounder when underfed or overfed. They stated that the amount of energy allocated for the development of the immune system depends on the body’s energy budget. Therefore, the animal body adapts to prevent the risk of starvation by increasing body reservations [66]. Further studies on the effects of feeding rate on the immune system of fish are required.

Optimizing the feeding rate in aquaculture is recognized as a major challenge in promoting fish growth and achieving economic efficiency. Increasing aquaculture productivity with fish growth and efficient use of feed are key aspects of fish production systems. Determination of the best-fit model through various regression analyses can be used as an effective tool to determine OFR. However, the OFR can vary depending on the selected measurement value (e.g., growth and/or immunity parameters). In the present study, the OFR required to increase productivity (such as WG) was estimated to be 2.4% BW/d. However, when considering feed efficiency (such as FCR), an OFR of 0.7% BW/d may ensure better economics. To ensure the robust immunity of fish (e.g., lysozyme), a 1.7% BW/d supply was found to be the OFR. Previous studies on fish species have generally focused on fish growth (e.g., SGR) to determine OFR [9,15,36,46,67]. However, our findings suggest that OFRs vary depending on the selected parameters and cannot be elucidated using existing interpretations. Our study results suggest that the OFR for maximum growth, as well as the OFR, can achieve optimal efficiency when rearing in terms of feed efficiency, nutrient deposition, and immunity. In other words, various factors must be comprehensively considered when deriving the optimum feeding rate, which suggests that a new perspective and approach to fish production is necessary.

## 5. Conclusions

Taken together, the feeding rate significantly influenced the growth performance of starry flounder, whereas the final body weight, weight gain, specific growth rate, and thermal growth coefficient were higher at 2.0–3.2% BW/d. Whole-body and carcass crude protein and lipid contents were higher at 2.0–3.2% BW/d, indicating increased body reserves for energy and nutrients. Underfeeding (0.4% BW/day) reduced plasma triglyceride, cholesterol, and total protein levels. Lysozyme activity, which is a key enzyme fighting pathogenic bacteria, decreased in the plasma of fish fed at low feeding rates (0.4–1.2% BW/d). Corresponding to these results, the OFR from a weight gain perspective was 2.4% BW/d, according to the quadratic broken-line model, which was recognized as the best-fit model. These findings provide a basis for nutritional optimization of starry flounder and in-depth studies on animal nutrition and physiology. Further studies are required to determine the optimum feeding rates at different life stages and the mechanisms underlying the effects of feeding rates on the fish immune system.

## Figures and Tables

**Table 1:** Growth and biological indices of starry flounder fed at various feeding rates for 10 weeks.

Measurement	Feeding Rate (% Body Weight/Day)								*p*-Value
	0.4%	0.8%	1.2%	1.6%	2.0%	2.4%	2.8%	3.2%	
Growth performance									
Survival, %	100.0 ± 0.0	100.0 ± 0.0	100.0 ± 0.0	100.0 ± 0.0	100.0 ± 0.0	100.0 ± 0.0	100.0 ± 0.0	100.0 ± 0.0	-
FBW, g/fish	220.4 ± 1.7 ^e^	279.9 ± 1.2 ^d^	337.6 ± 2.8 ^c^	371.6 ± 5.4 ^b^	388.1 ± 1.3 ^a^	400.6 ± 3.5 ^a^	400.9 ± 7.2 ^a^	400.2 ± 9.1 ^a^	<0.001
WG, %	20.1 ± 0.9 ^e^	52.5 ± 0.6 ^d^	83.9 ± 1.5 ^c^	102.4 ± 2.9 ^b^	111.4 ± 0.7 ^a^	118.2 ± 1.9 ^a^	118.4 ± 3.9 ^a^	118.0 ± 4.9 ^a^	<0.001
SGR, %/day	0.3 ± 0.0 ^e^	0.7 ± 0.0 ^d^	1.0 ± 0.0 ^c^	1.2 ± 0.0 ^b^	1.3 ± 0.0 ^a^	1.3 ± 0.0 ^a^	1.3 ± 0.0 ^a^	1.3 ± 0.0 ^a^	<0.001
TGC	0.4 ± 0.0 ^e^	0.9 ± 0.0 ^d^	1.4 ± 0.0 ^c^	1.6 ± 0.0 ^b^	1.7 ± 0.0 ^a^	1.8 ± 0.0 ^a^	1.8 ± 0.0 ^a^	1.8 ± 0.0 ^a^	<0.001
FI, g/fish	40.5 ± 1.1 ^h^	86.7 ± 0.6 ^g^	154.0 ± 1.6 ^f^	206.8 ± 3.4 ^e^	286.3 ± 1.0 ^d^	347.3 ± 3.2 ^c^	412.9 ± 7.9 ^b^	454.9 ± 9.9 ^a^	<0.001
FCR	1.1 ± 0.1^e^	0.9 ± 0.0 ^f^	1.0 ± 0.0 ^f^	1.1 ± 0.0 ^e^	1.4 ± 0.0 ^d^	1.6 ± 0.0 ^c^	1.9 ± 0.1 ^b^	2.1 ± 0.1 ^a^	<0.001
Biological indices									
CF, g/cm^3^	1.6 ± 0.1 ^c^	1.7 ± 0.1 ^bc^	1.8 ± 0.1 ^abc^	2.0 ± 0.0 ^b^	2.0 ± 0.0 ^b^	1.9 ± 0.2 ^b^	2.1 ± 0.1 ^a^	1.9 ± 0.1 ^b^	<0.001
HSI, %	1.7 ± 0.1 ^d^	2.9 ± 0.3 ^c^	3.6 ± 0.2 ^bc^	4.2 ± 0.4 ^b^	4.5 ± 0.4 ^a^	4.4 ± 0.3 ^b^	4.7 ± 0.3 ^a^	4.3 ± 0.2 ^b^	<0.001
VSI, %	2.0 ± 0.2	2.0 ± 0.1	2.2 ± 0.1	2.0 ± 0.1	2.0 ± 0.1	2.0 ± 0.1	2.1 ± 0.1	2.0 ± 0.1	0.496

FBW = final body weight; WG = weight gain; SGR = specific growth rate; TGC = thermal growth coefficient; FI = feed intake; FCR = feed conversion ratio; CF = condition factor; HSI = hepatosomatic index; VSI = viscerosomatic index. Values (mean ± standard error of triplication) in the same row with different superscript letters are significantly different (Tukey’s HSD test, *p* < 0.05), and the absence of superscript letters indicates no significant difference (*p* > 0.05).

**Table 2:** Proximate composition (%, wet-matter basis) of the whole body, carcass, liver, and viscera of starry flounder fed at the various feeding rates for 10 weeks.

Measurement	Feeding Rate (% Body Weight/Day)								*p*-Value
	0.4%	0.8%	1.2%	1.6%	2.0%	2.4%	2.8%	3.2%	
Whole-body ^1^, %									
Moisture	71.2 ± 0.1	69.5 ± 0.5	70.3 ± 0.2	69.3 ± 0.3	69.0 ± 0.4	70.6 ± 0.0	69.4 ± 0.1	68.8 ± 0.4	0.060
Crude protein	18.4 ± 0.2	18.4 ± 0.1	17.9 ± 0.2	18.6 ± 0.2	18.1 ± 0.2	17.7 ± 0.2	18.0 ± 0.1	18.2 ± 0.3	0.596
Crude lipid	5.2 ± 0.1 ^c^	7.3 ± 0.3 ^b^	7.5 ± 0.1 ^ab^	8.3 ± 0.3 ^ab^	8.8 ± 0.1 ^a^	7.7 ± 0.1 ^ab^	8.4 ± 0.1 ^ab^	8.8 ± 0.2 ^a^	<0.001
Crude ash	4.1 ± 0.0 ^a^	3.9 ± 0.2 ^a^	3.3 ± 0.0 ^b^	3.2 ± 0.0 ^b^	3.3 ± 0.0 ^b^	3.0 ± 0.0 ^b^	3.0 ± 0.0 ^b^	3.1 ± 0.0 ^b^	<0.001
Carcass ^2^_,_ %									
Moisture	71.2 ± 0.1 ^a^	70.4 ± 0.1 ^ab^	69.3 ± 0.4 ^ab^	68.8 ± 0.3 ^abc^	67.9 ± 0.2 ^bc^	66.4 ± 0.6 ^c^	66.7 ± 0.2 ^c^	66.4 ± 0.0 ^c^	<0.001
Crude protein	18.8 ± 0.1 ^b^	18.8 ± 0.2 ^b^	19.1 ± 0.1 ^ab^	19.7 ± 0.1 ^ab^	19.5 ± 0.1 ^ab^	19.3 ± 0.2 ^ab^	20.0 ± 0.3 ^ab^	20.6 ± 0.1 ^a^	0.010
Crude lipid	5.2 ± 0.2 ^c^	7.2 ± 0.1 ^ab^	6.9 ± 0.2 ^bc^	7.5 ± 0.1 ^ab^	7.9 ± 0.4 ^ab^	8.3 ± 0.2 ^ab^	9.0 ± 0.1 ^a^	8.6 ± 0.1 ^ab^	<0.001
Crude ash	4.6 ± 0.1 ^a^	4.3 ± 0.1 ^ab^	4.0 ± 0.1 ^abc^	3.6 ± 0.1 ^bc^	3.5 ± 0.0 ^c^	3.3 ± 0.1 ^c^	3.6 ± 0.0 ^bc^	3.8 ± 0.1 ^bc^	<0.001
Liver ^3^, %									
Moisture	70.3 ± 0.4 ^a^	65.5 ± 0.7 ^b^	62.1 ± 0.6 ^bc^	58.4 ± 0.2 ^cd^	56.7 ± 0.2 ^d^	59.1 ± 0.8 ^cd^	58.1 ± 0.8 ^cd^	57.9 ± 0.5 ^cd^	<0.001
Crude protein	10.2 ± 0.2 ^a^	8.4 ± 0.1 ^b^	8.0 ± 0.1 ^bc^	7.5 ± 0.0 ^cd^	7.3 ± 0.1 ^cd^	7.3 ± 0.1 ^cd^	7.0 ± 0.1 ^d^	7.3 ± 0.0 ^cd^	<0.001
Crude lipid	8.0 ± 0.4 ^d^	11.1 ± 1.0 ^cd^	13.9 ± 0.3 ^abcd^	13.4 ± 0.1 ^bcd^	20.2 ± 0.4 ^a^	17.3 ± 1.0 ^abc^	18.4 ± 1.4 ^ab^	19.7 ± 0.5 ^ab^	<0.001
Crude ash	1.7 ± 0.0 ^ab^	1.8 ± 0.1 ^a^	1.5 ± 0.1 ^abc^	1.1 ± 0.1 ^bc^	1.7 ± 0.1 ^ab^	1.4 ± 0.0 ^abc^	1.1 ± 0.1 ^c^	1.8 ± 0.0 ^a^	0.002
Viscera ^4^, %									
Moisture	82.7 ± 0.3	81.1 ± 0.2	81.8 ± 0.3	81.5 ± 0.3	80.7 ± 0.2	81.6 ± 0.0	82.2 ± 0.1	80.6 ± 0.3	0.062
Crude protein	13.5 ± 0.3	14.4 ± 0.2	13.8 ± 0.2	14.0 ± 0.3	14.5 ± 0.1	13.9 ± 0.0	13.4 ± 0.1	14.2 ± 0.2	0.237
Crude lipid	1.2 ± 0.0 ^e^	1.5 ± 0.0 ^de^	1.5 ± 0.1 ^cde^	1.9 ± 0.1 ^ab^	2.2 ± 0.0 ^a^	1.9 ± 0.0 ^ab^	1.7 ± 0.1 ^bcd^	1.9 ± 0.0 ^abc^	<0.001
Crude ash	1.2 ± 0.0	1.2 ± 0.0	1.3 ± 0.0	1.3 ± 0.0	1.3 ± 0.0	1.3 ± 0.0	1.2 ± 0.0	1.3 ± 0.0	0.128

Values (mean ± standard error of triplication) in the same row with different superscript letters are significantly different (Tukey’s HSD test, *p* < 0.05), and no letter superscript indicates no significant difference (*p* > 0.05). ^1^ Initial whole-body proximate composition (%) was moisture 74.7 ± 0.1, crude protein 16.0 ± 0.2, crude lipid 5.3 ± 0.1, and crude ash 3.4 ± 0.2, respectively. ^2^ Initial carcass proximate composition (%) was moisture 73.7 ± 0.4, crude protein 16.8 ± 0.4, crude lipid 4.8 ± 0.2, and crude ash 4.2 ± 0.3. ^3^ Initial liver proximate composition (%) was moisture 71.4 ± 0.4, crude protein 10.2 ± 0.2, crude lipid 12.1 ± 1.1, and crude ash 1.3 ± 0.1. ^4^ Initial viscera proximate composition (%) was moisture 84.1 ± 0.6, crude protein 13.3 ± 0.2, crude lipid 1.2 ± 0.1, and crude ash 1.2 ± 0.0.

**Table 3:** Plasma metabolites (24 h postprandial) of starry flounder fed at the various feeding rates for 10 weeks ^1^.

Measurement	Feeding Rate (% Body Weight/Day)								*p*-Value
	0.4%	0.8%	1.2%	1.6%	2.0%	2.4%	2.8%	3.2%	
AST, U/L	14.9 ± 2.4	23.1 ± 1.3	21.5 ± 0.5	23.2 ± 3.6	26.8 ± 2.8	23.4 ± 1.8	22.6 ± 3.2	25.0 ± 5.1	0.262
ALT, U/L	8.0 ± 2.3	11.5 ± 2.2	12.7 ± 2.5	12.8 ± 4.0	15.8 ± 3.2	13.1 ± 2.7	13.1 ± 1.9	14.1 ± 2.9	0.725
Triglycerides, mg/dL	34.0 ± 6.5 ^d^	57.9 ± 6.5 ^cd^	91.0 ± 15.9 ^bcd^	154.4 ± 33.9 ^ab^	131.0 ± 8.6 ^abc^	125.9 ± 12.9 ^abc^	131.7 ± 23.6 ^abc^	193.5 ± 18.0 ^a^	<0.001
Cholesterol, mg/dL	203.9 ± 7.7 ^b^	276.0 ± 27.9 ^a^	259.8 ± 11.7 ^ab^	282.6 ± 3.5 ^a^	268.0 ± 3.6 ^a^	267.9 ± 8.4 ^a^	294.3 ± 5.7 ^a^	277.3 ± 4.4 ^a^	0.003
Glucose, mmol/L	32.1 ± 4.3	37.3 ± 6.6	38.7 ± 9.3	40.5 ± 4.1	50.6 ± 5.5	47.5 ± 6.2	52.0 ± 2.3	44.8 ± 7.0	0.294
Total proteins, g/dL	3.3 ± 0.1 ^c^	4.1 ± 0.4 ^ab^	4.0 ± 0.2 ^bc^	4.6 ± 0.0 ^ab^	4.6 ± 0.0 ^ab^	4.3 ± 0.1 ^ab^	4.8 ± 0.0 ^a^	4.6 ± 0.0 ^ab^	<0.001

AST = aspartate aminotransferase; ALT = alanine aminotransferase. Values (mean ± standard error of triplication; five pooled fish per tank) with different superscripts within each row are significantly different (*p* < 0.05) according to Tukey’s HSD test.

**Table 4:** Protein and lipid gains and retentions in the whole body, carcass, liver, and viscera of starry flounder fed at various feeding rates for 10 weeks.

Measurement	Feeding Rate (% Body Weight/Day)								*p*-Value
	0.4%	0.8%	1.2%	1.6%	2.0%	2.4%	2.8%	3.2%	
Composition of gain, g									
Whole-body ^1^ protein	0.11 ± 0.00 ^d^	0.22 ± 0.00 ^c^	0.31 ± 0.01 ^b^	0.40 ± 0.01 ^a^	0.41 ± 0.01 ^a^	0.42 ± 0.01 ^a^	0.43 ± 0.01 ^a^	0.43 ± 0.01 ^a^	<0.001
Whole-body lipid	0.02 ± 0.00 ^d^	0.11 ± 0.01 ^c^	0.16 ± 0.00 ^c^	0.21 ± 0.01 ^b^	0.24 ± 0.00 ^ab^	0.21 ± 0.01 ^ab^	0.24 ± 0.00 ^ab^	0.25 ± 0.01 ^a^	<0.001
Carcass ^2^ protein	0.11 ± 0.00 ^f^	0.22 ± 0.01 ^e^	0.34 ± 0.00 ^d^	0.42 ± 0.00 ^c^	0.45 ± 0.01 ^c^	0.46 ± 0.01 ^bc^	0.49 ± 0.01 ^ab^	0.51 ± 0.00 ^a^	<0.001
Carcass lipid	0.03 ± 0.00 ^e^	0.11 ± 0.00 ^d^	0.15 ± 0.01 ^cd^	0.19 ± 0.00 ^bc^	0.22 ± 0.02 ^ab^	0.25 ± 0.01 ^ab^	0.27 ± 0.00 ^a^	0.26 ± 0.00 ^a^	<0.001
Liver ^3^ protein	0.04 ± 0.00 ^b^	0.05 ± 0.00 ^b^	0.08 ± 0.00 ^a^	0.09 ± 0.00 ^a^	0.10 ± 0.00 ^a^	0.11 ± 0.00 ^a^	0.10 ± 0.00 ^a^	0.11 ± 0.00 ^a^	<0.001
Liver lipid	−0.05 ± 0.01 ^d^	0.09 ± 0.03 ^cd^	0.25 ± 0.01 ^bc^	0.27 ± 0.00 ^bc^	0.56 ± 0.02 ^a^	0.47 ± 0.04 ^ab^	0.51 ± 0.05 ^a^	0.56 ± 0.02 ^a^	<0.001
Viscera ^4^ protein	0.05 ± 0.01 ^d^	0.16 ± 0.01 ^c^	0.22 ± 0.01 ^b^	0.27 ± 0.01 ^ab^	0.32 ± 0.00 ^a^	0.31 ± 0.00 ^a^	0.29 ± 0.01 ^a^	0.32 ± 0.00 ^a^	<0.001
Viscera lipid	0.005 ± 0.001 ^d^	0.019 ± 0.001 ^cd^	0.029 ± 0.003 ^bc^	0.049 ± 0.002 ^ab^	0.064 ± 0.001 ^a^	0.054 ± 0.001 ^a^	0.048 ± 0.003 ^ab^	0.053 ± 0.001 ^a^	<0.001
Nutrient retention (%)									
Whole-body protein	51.4 ± 2.0 ^a^	46.2 ± 0.5 ^ab^	39.2 ± 0.8 ^bc^	34.5 ± 0.5 ^cd^	27.3 ± 0.5 ^de^	22.5 ± 0.4 ^ef^	19.9 ± 0.3 ^f^	17.6 ± 0.3 ^f^	<0.001
Whole-body lipid	29.1 ± 3.9 ^c^	86.6 ± 5.9 ^a^	76.3 ± 1.1 ^a^	70.7 ± 3.5 ^a^	62.9 ± 0.6 ^ab^	44.6 ± 1.3 ^bc^	42.7 ± 0.7 ^bc^	40.0 ± 0.8 ^bc^	<0.001
Carcass protein	48.8 ± 1.0 ^a^	45.2 ± 1.1 ^ab^	42.7 ± 0.3 ^b^	36.8 ± 0.3 ^c^	29.9 ± 0.4 ^d^	25.0 ± 0.4 ^de^	22.9 ± 0.3 ^e^	20.8 ± 0.2 ^e^	<0.001
Carcass lipid	46.7 ± 6.0 ^bc^	91.2 ± 2.3 ^a^	71.7 ± 3.7 ^ab^	63.9 ± 1.6 ^bc^	56.4 ± 4.4 ^bc^	51.4 ± 2.1 ^bc^	48.6 ± 0.6 ^bc^	40.3 ± 0.6 ^c^	<0.001
Liver protein	17.6 ± 1.3 ^a^	9.7 ± 0.8 ^b^	10.4 ± 0.5 ^b^	8.0 ± 0.2 ^bc^	6.5 ± 0.2 ^bc^	5.7 ± 0.2 ^bc^	4.4 ± 0.2 ^c^	4.3 ± 0.1 ^c^	<0.001
Liver lipid	−81.6 ± 16.5 ^b^	72.0 ± 22.7 ^a^	122.0 ± 5.2 ^a^	92.5 ± 0.5 ^a^	145.6 ± 4.1 ^a^	99.1 ± 9.1 ^a^	92.4 ± 9.5 ^a^	88.7 ± 3.2 ^a^	<0.001
Viscera protein	25.0 ± 3.1 ^abc^	33.0 ± 1.2 ^a^	28.1 ± 1.0 ^ab^	23.9 ± 1.1 ^abcd^	21.2 ± 0.2 ^bcd^	16.9 ± 0.1 ^cd^	13.6 ± 0.2 ^d^	13.2 ± 0.1 ^d^	<0.001
Viscera lipid	8.2 ± 1.6 ^b^	15.7 ± 0.5 ^a^	14.1 ± 1.3 ^ab^	16.6 ± 0.7 ^a^	16.7 ± 0.2 ^a^	11.2 ± 0.2 ^ab^	8.6 ± 0.5 ^b^	8.4 ± 0.2 ^b^	0.001

Values (mean ± standard error of triplication) with different superscripts within each row are significantly different (*p* < 0.05), according to Tukey’s HSD test. Gain and nutrient retention were calculated using the following equations: [(FBW or FTW × FNC) − (IBW or ITW× INC)]/100 and [(FBW or FTW × FNC) − (IBW or ITW × INC)]/nutrient feed (g), where FBW, FTW, IBW, and ITW are the final body weight (g), final tissue weight (g), initial body weight (g) and initial tissue weights (g), respectively, and FNC and INC are the final and initial nutrient compositions (%) in the body and tissue, respectively. ^1^ Initial whole-body proximate composition (%) was moisture 74.7 ± 0.1, crude protein 16.0 ± 0.2, crude lipid 5.3 ± 0.1, and crude ash 3.4 ± 0.2, respectively. ^2^ Initial carcass proximate composition (%) was moisture 73.7 ± 0.4, crude protein 16.8 ± 0.4, crude lipid 4.8 ± 0.2, and crude ash 4.2 ± 0.3. ^3^ Initial liver proximate composition (%) was moisture 71.4 ± 0.4, crude protein 10.2 ± 0.2, crude lipid 12.1 ± 1.1, and crude ash 1.3 ± 0.1. ^4^ Initial viscera proximate composition (%) was moisture 84.1 ± 0.6, crude protein 13.3 ± 0.2, crude lipid 1.2 ± 0.1, and crude ash 1.2 ± 0.0.

**Table 5:** Estimated feeding rates on the variety of measurements through the regression analyses, including one-slope straight broken-line model (One-slope BL), two-slope straight broken-line model (Two-slope BL), quadratic broken-line model (Quadratic BL), and second-order polynomial model (Quadratic) in starry flounder fed at the various feeding rate.

Measurement	Optimum Feeding Rate (%)				Measurement	Optimum Feeding Rate (%)			
	One-Slope BL	Two-Slope BL	Quadratic BL	Quadratic		One-Slope BL	Two-Slope BL	Quadratic BL	Quadratic
Growth Performance					Composition of Gain (g)				
FBW ^1^ (g)	1.7	1.7	***2.4*** ^22^	2.6	WB ^9^ protein	1.7	***1.5***	2.3	2.6
WG ^2^ (%)	1.7	1.7	***2.4***	2.6	WB lipid	1.7	1.6	***2.3***	2.7
SGR ^3^ (%)	1.7	1.6	***2.2***	2.5	Carcass protein	2.0	***1.5***	2.7	2.9
TGC ^4^	1.7	1.6	***2.3***	2.6	Carcass lipid	2.2	2.2	***3.1***	***3.1***
FCR ^5^	NA ^23^	NA	NA	***0.7***	Liver protein	***1.7***	1.7	2.5	2.7
Biological indices					Liver lipid	***2.0***	2.0	3.2	3.2
CF ^6^	1.6	2.0	2.3	***2.5***	Viscera protein	1.8	1.7	***2.3***	2.6
HSI ^7^	1.5	1.7	***2.1***	2.5	Viscera lipid	1.8	***2.0***	2.3	2.5
VSI ^8^	NS ^24^	NS	NS	NS	Nutrient retention (%)				
Whole-body proximate composition (%; wet-matter basis)					WB protein	***2.6***	***2.3***	4.9	4.9
Moisture	NS	NS	NS	NS	WB lipid	NA	NA	NA	***1.6***
Protein	NS	NS	NS	NS	Carcass protein	***2.7***	***2.5***	9.6	9.6
Lipid	1.4	1.3	***1.8***	2.5	Carcass lipid	NA	***0.8***	NA	NS
Ash	2.1	2.1	***2.4***	2.7	Liver protein	2.2	1.6	***2.9***	3.0
Carcass proximate composition (%; wet-matter basis)					Liver lipid	***0.9***	1.7	***1.1***	2.1
Moisture	***2.5***	2.4	NS	NS	Viscera protein	NA	***0.8***	NA	NS
Protein	NA	NS	NA	NS	Viscera lipid	NA	1.7	NA	***1.6***
Lipid	***2.4***	2.3	NS	NS	Plasma metabolites				
Ash	1.8	2.2	NS	***2.3***	AST ^10^	NS	NS	NS	NS
Liver proximate composition (%; wet-matter basis)					ALT ^11^	NS	NS	NS	NS
Moisture	***1.6***	1.6	2.1	2.5	TG ^12^	1.6	***1.6***	NS	NS
Protein	1.4	***0.9***	1.8	2.4	CHOL ^13^	NA	NA	NA	***2.4***
Lipid	***2.0***	2.0	3.2	3.2	GLU ^14^	NS	NS	NS	NS
Ash	NS	NA	NS	NS	TP ^15^	1.6	1.6	***2.0***	2.6
Viscera proximate composition (%; wet-matter basis)					Innate immunity in plasma				
Moisture	NS	NS	NA	NS	LYZ ^16^	1.6	***1.7***	2.1	2.3
Protein	NS	NS	NA	NS	IL-1β ^17^	NS	NS	NS	NS
Lipid	1.8	***2.0***	NS	2.3	IgM ^18^	NS	NS	NS	NS
Ash	NS	***2.0***	NS	NS	GPX ^19^	NS	NS	NS	NS
					SOD ^20^	NS	NS	NS	NS
					HSP70 ^21^	NS	NS	NS	NS

^1^ Final body weight (g/fish); ^2^ Weight gain (%); ^3^ Specific growth rate (%/day); ^4^ Thermal growth coefficient; ^5^ Feed conversion ratio; ^6^ Condition factor (g/cm^3^); ^7^ Hepatosomatic index (%); ^8^ Viscerosomatic index (%); ^9^ Whole body; ^10^ Aspartate aminotransferase (U/L);^11^ Alanine aminotransferase (U/L); ^12^ Triglycerides (mg/dL); ^13^ Cholesterol (mg/dL); ^14^ Glucose (mmol/L); ^15^ Total proteins (g/dL); ^16^ Lysozyme (ng/mL); ^17^ Interleukin 1β (pg/mL); ^18^ Immunoglobulin M (µg/mL); ^19^ Glutathione peroxidase (µg/mL); ^20^ Superoxide dismutase (ng/mL); ^21^ Heat shock protein 70 (pg/mL); ^22^ Bold and italic numbers indicate the best estimate among the estimated feeding rates by the regression analyses based on model selection criteria, including adjusted coefficient correlation and corrected Akaike information criterion. ^23^ Not available: The tested model was not able to estimate the optimum feeding rate due to failure of the estimation algorithm to achieve convergence. ^24^ Not significant: The coefficient of one or more variables was not statistically different from zero.

## Data Availability

Data will be available from the corresponding authors by reasonable request. An earlier version of the manuscript was presented as a Preprint [68] https://doi.org/10.2139/ssrn.4783733.

## Supplementary Materials

The supplementary files are available online at https://www.mdpi.com/article/10.3390/ani14213127/s1. The supplementary file shows the estimated feeding rates on a variety of measurements through the regression analyses, including four models, AICc and R^2^adj in starry flounder fed at various feeding rates.
