# Supplementary material for: Growth, Nutrient Deposition, Plasma Metabolites, and Innate Immunity Are Associated with Feeding Rate in Juvenile Starry Flounder (Platichthys stellatus)
Source: Animals (Basel). 2024 Oct 30;14(21):3127. doi: 10.3390/ani14213127 (PMC11544839; doi:10.3390/ani14213127)
Supplement: Supplementary file 1 [file animals-14-03127-s001.zip › animals-3264133-supplementary.pdf]

Supplementary Table. Optimum feeding rate (OFR), R^2_{adj} and AICc of regression analyses, including one-slope straight broken-line model (One-slope BL), two-slope straight broken-line model (Two-slope BL), quadratic broken-line model (Quadratic BL), and second-order polynomial model (Quadratic) in starry flounder fed at the various feeding rate

Measurement	One-slope BL			Two-slope BL			Quadratic BL			Quadratic		
	OFR (%)	R^2_{adj}	AICc	OFR (%)	R^2_{adj}	AICc	OFR (%)	R^2_{adj}	AICc	OFR (%)	R^2_{adj}	AICc
Growth performance												
FBW[1] (g)	1.7	0.9844	172.8	1.7	0.9861	171.7	***2.4***[22]	***0.9937***	***151.2***	2.6	0.9897	162.9
WG[2] (%)	1.7	0.9844	143.7	1.7	0.9861	142.5	***2.4***	***0.9937***	***122***	2.6	0.9897	133.7
SGR[3] (%)	1.7	0.9795	-67.8	1.6	0.9808	-66.5	***2.2***	***0.9945***	***-102.9***	2.5	0.9817	-73.9
TGC[4]	1.7	0.98	-53.8	1.6	0.9809	-53.1	***2.3***	***0.9945***	***-84.9***	3.6	0.9854	-61.3
FCR[5]	NA[23]	NA	NA	NA	NA	NA	NA	NA	NA	***0.7***	***0.9665***	***-50.2***
Biological indices												
CF[6]	1.6	0.6464	-41.6	2.0	0.6015	-37	2.3	0.6385	-41	***2.5***	***0.658***	***-42.4***
HSI[7]	1.5	0.9113	14.5	1.7	0.9059	17.6	***2.1***	***0.925***	***10.4***	2.5	0.9195	12.1
VSI[8]	NS[24]	NS	NS	NS	NS	NS	NS	NS	NS	NS	NS	NS
Whole-body proximate composition (%; wet-matter basis)												
Moisture	NS	NS	NS	NS	NS	NS	NS	NS	NS	NS	NS	NS
Protein	NS	NS	NS	NS	NS	NS	NS	NS	NS	NS	NS	NS
Lipid	1.4	0.7153	51.5	1.3	0.7055	54	***1.8***	***0.7457***	***48.8***	2.5	0.67	55.1
Ash	2.1	0.724	0.68	2.1	0.7098	3.6	***2.4***	***0.7768***	***-4.4***	2.7	0.7725	-3.96
Carcass proximate composition (%; wet-matter basis)												
Moisture	***2.5***	***0.8206***	***62.8***	2.4	0.8125	65.6	NS	NA	NA	NS	NA	NA
Protein	NA	NA	NA	NS	NA	NA	NA	NA	NA	NS	NA	NA
Lipid	***2.4***	***0.6551***	***58.7***	2.3	0.6411	61.3	NS	NA	NA	NS	NA	NA
Ash	1.8	0.659	9.8	2.2	0.7077	7.8	NS	NA	NA	***2.3***	***0.688***	***7.6***
Liver proximate composition (%; wet-matter basis)												
Moisture	***1.6***	***0.8732***	***97.8***	1.6	0.8668	100.6	2.1	0.87	98.4	2.5	0.8475	102.2
Protein	1.4	0.8689	25.1	***0.9***	***0.9104***	***17.7***	1.8	0.9	18.6	2.4	0.8644	25.9
Lipid	***2.0***	***0.7069***	***116.7***	2.0	0.6958	119.3	3.2	0.6844	118.5	3.2	0.6844	118.5
Ash	NS	NA	NA	NA	NA	NA	NS	NA	NA	NS	NA	NA
Viscera proximate composition (%; wet-matter basis)												
Moisture	NS	NA	NA	NS	NA	NA	NA	NA	NA	NS	NA	NA
Protein	NS	NA	NA	NS	NA	NA	NA	NA	NA	NS	NA	NA
Lipid	1.8	0.6422	-5.7	***2.0***	***0.727***	***-10.5***	NS	NA	NA	2.3	0.6253	-4.6
Ash	NS	NA	NA	***2.0***	***0.0772***	***-88.3***	NS	NA	NA	NS	NA	NA
Composition of gain (g)												
WB[9] protein	1.7	0.9706	-116.1	***1.5***	***0.9747***	***-118.1***	2.3	0.9706	-116.1	2.6	0.9607	-109.2
WB lipid	1.7	0.9309	-112.5	1.6	0.9316	-111.1	***2.3***	***0.937***	***-114.7***	2.7	0.9173	-108.2
Carcass protein	2.0	0.953	-95.2	***1.5***	***0.9894***	***-129.2***	2.7	0.9779	-113.3	2.9	0.9753	-110.6
Carcass lipid	2.2	0.9011	-102.4	2.2	0.8973	-99.8	***3.1***	***0.9196***	***-107.4***	***3.1***	***0.9197***	***-107.4***
Liver protein	***1.7***	***0.8394***	***-145.6***	1.7	0.833	-143	2.5	0.8336	-144.8	2.7	0.8244	-143.5
Liver lipid	***2.0***	***0.8471***	***-42.4***	2.0	0.845	-40.4	3.2	0.8279	-39.6	3.2	0.8279	-39.6
Viscera protein	1.8	0.9432	-110.1	1.7	0.9402	-107.2	***2.3***	***0.9526***	***-114.4***	2.6	0.9361	-107.3
Viscera lipid	1.8	0.8853	-167.1	***2.0***	***0.9104***	***-171.4***	2.3	0.8524	-161	2.5	0.8516	-161

Nutrient retention (%)												
WB protein	***2.6***	***0.9601***	***115.6***	***2.3***	***0.9611***	***116.7***	4.9	0.9582	116.7	4.9	0.9582	116.7
WB lipid	NA	NA	NA	NA	NA	NA	NA	NA	NA	***1.6***	***0.3387***	***NS***
Carcass protein	***2.7***	***0.9606***	***108***	***2.5***	***0.9615***	***109.1***	9.6	0.9531	112.2	9.6	0.9531	112.2
Carcass lipid	NA	NA	NS	***0.8***	***0.575***	***NS***	NA	NA	NS	NS	NA	NS
Liver protein	2.2	0.7452	111.5	1.6	0.7533	112.4	***2.9***	***0.7835***	***107.58***	3.0	0.7829	107.65
Liver lipid	***0.9***	***0.7464***	***244.9***	1.7	0.5348	261.1	***1.1***	***0.7464***	***244.9***	2.1	0.6076	255.4
Viscera protein	NA	NS	NS	***0.8***	***0.7397***	***NS***	NA	NS	NS	NS	NS	NS
Viscera lipid	NA	NS	NS	1.7	0.5056	125.9	NA	NS	NS	***1.6***	***0.4882***	***125.1***
Plasma metabolites												
AST[10]	NS	NS	NS	NS	NS	NS	NS	NS	NS	NS	NS	NS
ALT[11]	NS	NS	NS	NS	NS	NS	NS	NS	NS	NS	NS	NS
TG[12]	1.6	0.5821	245.2	***1.6***	***0.6046***	***245.5***	NS	NS	NS	NS	NS	NS
CHOL[13]	NA	NS	NS	NA	NS	NS	NA	NS	NS	***2.4***	***0.3427***	***228***
GLU[14]	NS	NS	NS	NS	NS	NS	NS	NS	NS	NS	NS	NS
TP[15]	1.6	0.6301	15.7	1.6	0.6132	18.5	***2.0***	***0.6347***	***15.4***	2.6	0.6102	17
Innate immunity in plasma												
LYZ[16]	1.6	0.8463	-72.7	***1.7***	***0.8772***	***-76.4***	2.1	0.7898	-65.1	2.3	0.8214	-69.1
IL-1β[17]	NS	NS	NS	NS	NS	NS	NS	NS	NS	NS	NS	NS
IgM[18]	NS	NS	NS	NS	NS	NS	NS	NS	NS	NS	NS	NS
GPX[19]	NS	NS	NS	NS	NS	NS	NS	NS	NS	NS	NS	NS
SOD[20]	NS	NS	NS	NS	NS	NS	NS	NS	NS	NS	NS	NS
HSP70[21]	NS	NS	NS	NS	NS	NS	NS	NS	NS	NS	NS	NS

[1]Final body weight (g/fish); [2]Weight gain (%); [3]Specific growth rate (%/day); [4]Thermal growth coefficient; [5]Feed conversion ratio; [6]Condition factor (g/cm^3); [7]Hepatosomatic index (%); [8]Viscerosomatic index (%); [9]Whole-body; [10]Aspartate aminotransferase (U/L);[11]Alanine aminotransferase (U/L); [12]Triglycerides (mg/dL); [13]Cholesterol (mg/dL); [14]Glucose (mmol/L); [15]Total proteins (g/dL); [16]Lysozyme (ng/mL); [17]Interleukin 1β (pg/mL); [18]Immunoglobulin M (µg/mL); [19]Glutathione peroxidase (µg/mL); [20]Superoxide dismutase (ng/mL); [21]Heat shock protein 70 (pg/mL)

[22]The bold and italic numbers indicate the regression model is the most suitable model to reflect the parameters of fish.

[23]Not available: The tested model was not able to estimate the optimum feeding rate due to failure of the estimation algorithm to achieve convergence.

[24]Not significant: The coefficient of one or more variables was not statistically different from zero.
